# The Therapeutic Effect of a Biodegradable Long-Acting Intravitreal Implant Containing CGK012 on Neovascular Age-Related Macular Degeneration by Promoting β-Catenin Degradation

**DOI:** 10.3390/ph18121884

**Published:** 2025-12-12

**Authors:** Seoyoung Park, Jihyun Won, Jong Beom Heo, Juhyung Kang, Ye Woon Oh, Geunji Park, Giseong Lee, Jee-Hyun Lee, Gyu-Yong Song, Wonku Kang, Sangtaek Oh

**Affiliations:** 1 Inspharmtech, Inc., Chuncheon 24341, Republic of Korea; 2 College of Pharmacy, Chung-Ang University, Seoul 06974, Republic of Korea; 3 College of Pharmacy, Chungnam National University, Daejeon 34134, Republic of Korea; 4 Department of Bio and Fermentation Convergence Technology, Kookmin University, Seoul 02707, Republic of Korea; 5 College of General Education, Kookmin University, Seoul 02707, Republic of Korea; 6 AREZ Co., Ltd., Daejeon 34036, Republic of Korea

**Keywords:** neovascular age-related macular degeneration, CGK012, intravitreal implant, Wnt/β-catenin pathway, angiogenic/inflammatory factors

## Abstract

**Background/Objectives:** Neovascular age-related macular degeneration (nAMD) poses a serious threat to the eyesight of older adults, representing a leading cause of irreversible vision loss. Anti-vascular endothelial growth factor (anti-VEGF) treatments are effective but require repeated intraocular injections and show poor responses in some patients. CGK012 is a novel derivative of decursin that inhibits the Wnt/β-catenin pathway. This study aimed to elucidate the mode of action of CGK012 and examine its therapeutic effects. **Methods:** We performed in vitro cellular studies in a retinal pigment epithelial (RPE) cell line (ARPE-19) and human umbilical vein endothelial cells (HUVECs). We examined the in vivo efficacy of CGK012-loaded implants in laser-induced choroidal neovascularization (CNV) rabbit models. We also determined the implants’ in vitro dissolution, intraocular release, and disposition characteristics. **Results:** CGK012 decreased angiogenic/proinflammatory factor expression and suppressed the epithelial–mesenchymal transition (EMT) in RPE cells by promoting intracellular β-catenin degradation. Additionally, it repressed the expression of cyclin D1 and c-myc, downstream target genes of β-catenin, and inhibited HUVEC capillary tube formation. CGK012-loaded poly (lactic-co-glycolic acid) (PLGA) intravitreal implants significantly reduced vascular leakage in a laser-induced CNV rabbit model. Notably, CGK012 released from the implant was highly permeable to retina/choroid tissue and downregulated β-catenin, angiogenic/inflammatory factors, and vimentin in the rabbit model. The CGK012 concentration reached a plateau at 28–42 days in the vitreous humor and decayed with a half-life of 14 days without systemic exposure. **Conclusions:** Our findings demonstrate that CGK012 implants prevent choroidal neovascularization through the Wnt/β-catenin pathway suppression and produce high concentrations of CGK012 in the posterior eye segment with prolonged release. Thus, these implants provide more therapeutic choices for nAMD treatment.

## 1. Introduction

Neovascular age-related macular degeneration (nAMD) leads to severe central vision impairment in older adults due to new blood vessel outgrowth towards the retinal pigment epithelium (RPE) or retinal degeneration caused by a rupture in the Bruch’s membrane from the choriocapillaris [1]. nAMD pathogenesis involves multiple factors, including oxidative imbalance, inflammation, hypoxic stress, and the dysregulated vascular endothelial growth factor (VEGF) [2,3,4]. At present, administering intraocular injections of anti-angiogenic agents that block VEGF activity can lead to significant visual improvement [5,6,7]. However, up to 30% patients with nAMD show inadequate response to anti-VEGF single-agent therapy, leading to refractory cases [8]. Moreover, repeated intravitreal injection of anti-VEGF can lead to severe side effects, including subconjunctival hemorrhage, retinal detachment, infectious endophthalmitis, and intraocular pressure elevation [9]. Thus, new therapeutic options are needed to reduce reliance on anti-VEGF treatments.

The Wnt/β-catenin pathway regulates various pathological processes, including angiogenesis, inflammation, and fibrosis [10,11,12]. Dysregulation of this pathway plays a pathogenic role in retinal neovascularization development. Decreased circulating Dickkopf-1 (DKK-1), a specific Wnt/β-catenin pathway inhibitor, is associated with nAMD development and severity [13]. The Wnt/β-catenin pathway is abnormally activated in the RPE in animal models for AMD [14]. Additionally, levels of LRP5/6 and β-catenin are increased in laser-induced choroidal neovascularization (CNV) models, commonly used for studying CNV in nAMD [15]. Moreover, constitutive expression of active β-catenin upregulates VEGF, nuclear factor-κB (NF-κB), and tumor necrosis factor-α (TNF-α) expression in human RPE cells and rat retinas [14]. Activation of Wnt/β-catenin promotes the epithelial–mesenchymal transition (EMT) and subsequent fibrosis in ARPE-19 cells [16], which may be pathogenic features in the RPE of AMD patients [17,18]. Therefore, the Wnt/β-catenin pathway is a potential target for nAMD therapeutic development.

Poly (lactic-co-glycolic acid) (PLGA) implants offer a promising drug delivery strategy to treat posterior segment eye diseases like nAMD, diabetic macular edema, and uveitis due to their ability to extend drug release, increase bioavailability, and decrease systemic adverse effects [19,20]. After administration, PLGA is enzymatically hydrolyzed into H_2_O and CO_2_ in the vitreous humor; therefore, implant removal is unnecessary. Several PLGA-based intravitreal implants have been approved for clinical application. For example, Surodex (Oculex Pharmaceuticals, Inc., Sunnyvale, CA, USA) and Ozurdex (Allergan, Inc., Dublin, Ireland) are used to deliver dexamethasone to the eye over several weeks to months, decreasing the intravitreal injection frequency [21].

Recently, we used a chemical biology approach to identify CGK012, a novel decursin derivative, as a Wnt/β-catenin pathway antagonist [22]. It promotes β-catenin degradation and also exhibits anti-inflammatory and antioxidant effects through the inhibition of NF-κB and activation of the Nrf2 pathways [22,23]. In this study, we show that CGK012-loaded PLGA intravitreal implants reduce laser-induced CNV by suppressing harmful β-catenin and allowing for long-term sustained release in the posterior segment of the eye.

## 2. Results

### 2.1. CGK012 Promotes Pathogenic β-Catenin Degradation in RPE Cells

**Figure 1:**
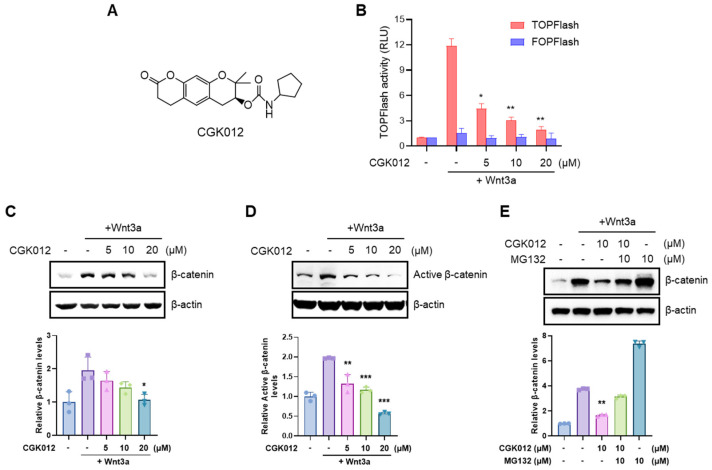
CGK012 suppresses the Wnt/β-catenin pathway in human ARPE-19 cells. (**A**) The chemical structure of CGK012. (**B**) CRT inhibition by CGK012 in ARPE-19 cells. Cells were transfected with reporter plasmids (TOPFlash or FOPFlash) for 24 h and then treated with CGK012 for 15 h. Luciferase reporter activities are determined as relative light units normalized to *Renilla reniformis* luciferase activity. The results are presented as the mean ± SD from three independent experiments. * *p* < 0.05, ** *p* < 0.01, compared with the Wnt3a-treated vehicle control group. (**C**) Cytosolic proteins were isolated from ARPE-19 cells treated with the vehicle (DMSO) or the indicated concentrations of CGK012 with or without Wnt3a-CM for 15 h. The samples were then analyzed using Western blot with an anti-β-catenin antibody. (**D**) Cells were incubated with various concentrations of CGK012 for 15 h and were then analyzed by Western blot with an antibody targeting active-β-catenin (non-phosphorylated β-catenin at Ser33/37/Thr41). Equal amounts of protein were loaded into each lane. (**E**) ARPE-19 cells treated with the vehicle or CGK012 (10 μM) in the presence of Wnt3a-CM and exposed to MG-132 (10 μM) for 8 h. β-catenin levels in the cytosolic fraction were determined by Western blot. β-actin (**C**–**E**) was used as the loading control. The levels of β-catenin were normalized to β-actin. The bar graphs represent the relative intensity of the quantified band. The results are presented as the mean ± SD of three independent experiments. * *p* < 0.05, ** *p* < 0.01, and *** *p* < 0.001, compared with the Wnt3a-treated vehicle control group.

β-catenin response transcription (CRT) was assessed in RPE cells to evaluate the effects of CGK012 on Wnt/β-catenin signaling, which are critically involved in the pathogenesis of nAMD [14]. ARPE-19 cells were transiently transfected with a synthetic β-catenin/TCF-dependent firefly luciferase reporter (TOPFlash), then treated with Wnt3a-CM and/or CGK012. ARPE-19 treatment with CGK012 led to a concentration-dependent decrease in CRT stimulated by Wnt3a-CM (Figure 1A,B). Conversely, the activity of FOPFlash, which contains a mutated TCF binding region, showed no significant changes by Wnt3a-CM and CGK012 (Figure 1B). Additionally, CGK012 reduced intracellular β-catenin and active β-catenin (non-phosphorylated β-catenin at Ser33/37/Thr41 residues) protein levels, which regulate CRT in ARPE-19 cells in the presence of Wnt3a-CM (Figure 1C,D). Consistent with a previous study [22], treatment with the proteasome inhibitor MG-132 abrogated CGK012-mediated β-catenin downregulation in ARPE-19 cells (Figure 1E), suggesting that CGK012 efficiently suppresses the Wnt/β-catenin pathway by facilitating the degradation of β-catenin in RPE cells.

### 2.2. CGK012 Inhibits the Pathogenic Effects of β-Catenin in RPE Cells

**Figure 2:**
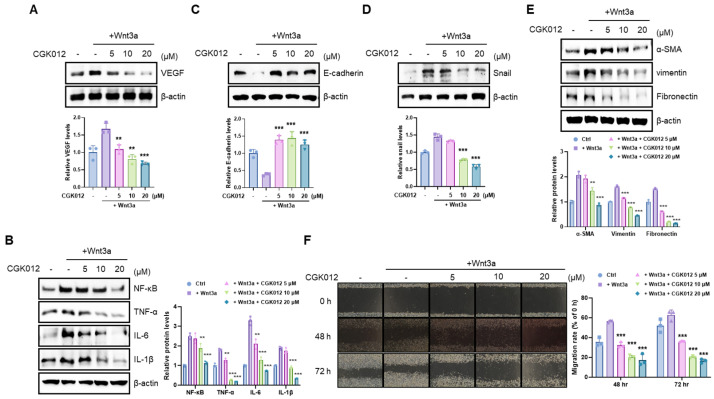
CGK012 reduced angiogenic/inflammatory factor expression in human ARPE-19 cells. (**A**–**E**). The levels of VEGF (**A**), NF-κB, TNFα, IL-6, and IL-1β (**B**), E-cadherin (**C**), Snail1 (**D**), and α-SMA, vimentin, and fibronectin (**E**) in whole cell lysates from ARPE-19 cells treated with CGK012 were detected using Western blot. In (**A**–**E**), β-actin was the loading control. The levels of protein expression were normalized to β-actin. The bar graph shows the average volume density corrected for the loading control. The results are presented as the mean ± SD of three independent experiments. (**F**) ARPE-19 cells were grown to confluence, scratched using 200-μL micro pipet tips, and incubated with Wnt3a-CM and/or CGK012. Representative images of cell migration obtained at 0, 48, and 72 h after treatment. The images were examined using an inverted microscope with a phase contrast at 4× magnification. The migration rates were measured using ImageJ software (version 1.54g). The bar graph represents the average migration rates adjusted for the 0 h control. The results are presented as the mean ± SD. ** *p* < 0.01, and *** *p* < 0.001, compared with the Wnt3a-treated vehicle control group.

Wnt/β-catenin pathway activation significantly increases angiogenic and proinflammatory factor expression in human RPE cells [14]. Given that CGK012 inhibits the Wnt/β-catenin pathway, we examined whether CGK012 represses angiogenic and proinflammatory factor expression. As presented in Figure 2A,B, in ARPE-19 cells treated with Wnt3a-CM, CGK012 reduced VEGF, NF-κB, TNF-α, IL-6, and IL-1β, well-characterized pathogenic factors in nAMD. Previous studies reported that Wnt/β-catenin signaling induces the EMT, impairing RPE structural integrity and causing retinal fibrosis [16]. Consistent with a previous report [24], in ARPE-19 cells, Wnt3a-CM significantly decreased the epithelial adhesion molecule E-cadherin, an established EMT characteristic (Figure 2C). However, CGK012 treatment abrogated the Wnt3a-CM effect on E-cadherin expression (Figure 2C). Conversely, Snail, an E-cadherin transcriptional repressor [25], increased in the presence of Wnt3a-CM (Figure 2D). Significantly, CGK012 inhibited the upregulation of Snail induced by Wnt3a in ARPE-19 cells (Figure 2D). Additionally, CGK012 treatment reduced fibrosis-related markers like α-smooth muscle actin, vimentin, and fibronectin caused by Wnt3a in ARPE-19 cells (Figure 2E). Subsequently, we examined the migration of ARPE-19 cells in the presence of Wnt3a-CM and/or CGK012. Consistently, Wnt3a-CM dramatically promoted ARPE-19 cell migration, whereas Wnt3a-CM and CGK012 coincubation inhibited cell migration (Figure 2F), indicating that CGK012 suppresses the Wnt3a-induced EMT in ARPE-19 cells.

### 2.3. CGK012 Inhibits the Wnt/β-Catenin Pathway and Capillary Tube Formation in HUVECs

**Figure 3:**
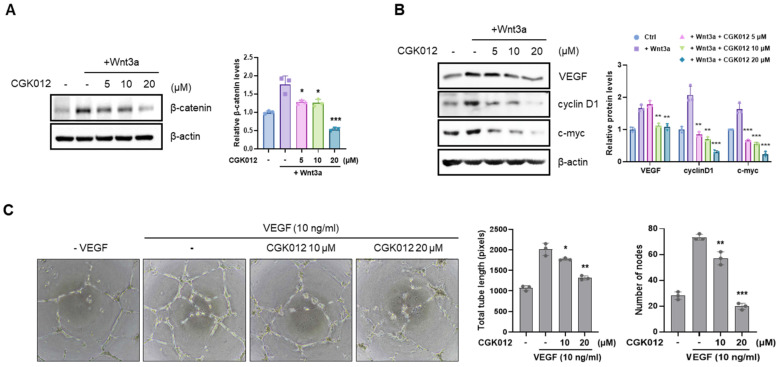
CGK012 suppresses the Wnt/β-catenin pathway in HUVECs. (**A**) The cytosolic fractions were prepared from HUVECs treated with Wnt3a-CM and/or CGK012 for 15 h. They were then analyzed by Western Blot with an antibody targeting β-catenin. (**B**) Total cell lysates were analyzed with VEGF, cyclin D1, and c-myc antibodies. In (**A**,**B**), β-actin was used as the loading control. The levels of protein expression were normalized to β-actin. The bar graphs represent the relative intensity of the quantified band. The results are presented as the mean ± SD of three independent experiments. * *p* < 0.05, ** *p* < 0.01, and *** *p* < 0.001, compared with the Wnt3a-treated vehicle control group. (**C**) The effect of CGK012 on VEGF-mediated capillary tube formation. The images were examined using an inverted microscope with a phase contrast at 4× magnification. The total tube length and the number of nodes were measured using ImageJ. The results are presented as the mean ± SD of three independent experiments. * *p* < 0.05, ** *p* < 0.01, and *** *p* < 0.001, compared to the VEGF-treated vehicle control group.

Next, we used Western Blot analysis to evaluate the CGK012 effect on cytosolic β-catenin levels in HUVECs. As presented in Figure 3A, CGK012 consistently decreased the intracellular β-catenin upregulated by Wnt3a-CM in HUVECs. We then examined the CGK012 effect on β-catenin-dependent gene expression in HUVECs. After HUVEC treatment with CGK012 at various concentrations, the expression of VEGF, cyclin D1, and c-myc, known downstream targets of β-catenin, was quantified by Western Blot analysis. Figure 3B shows the reduction in VEGF, cyclin D1, and c-myc protein levels in response to CGK012. As β-catenin overexpression augments angiogenesis by enhancing endothelial cell survival and inducing VEGF expression [26], we evaluated the effect of CGK012 on capillary tube formation in HUVECs. As shown in Figure 3C, HUVEC incubation with CGK012 decreased the formation of capillary tubes induced by VEGF in a dose-dependent manner.

### 2.4. In Vitro Release and Intraocular Profile of CGK012-Loaded PLGA Implant

**Figure 4:**
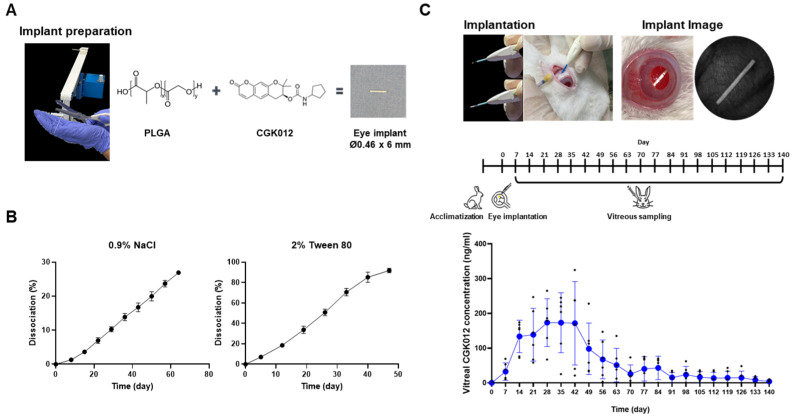
Biodegradable CGK012-loaded PLGA implants. (**A**) Schematic representation of PLGA implants loaded with CGK012. (**B**) In vitro dissolution profile of CGK012 implants (600 μg of CGK012) tested in normal saline with and without 2% Tween 80. (**C**) Ocular pharmacokinetic profile of CGK012 after intravitreal administration of implants (600 μg of CGK012) in NZW rabbits. Each dot represents a unique data point corrected from a single rabbit.

Biodegradable implants can release drugs directly into the vitreous and maintain a concentration in the therapeutic range long-term [27]. To study the potential for CGK012 long-term release, PLGA implants loaded with CGK012 were prepared as rod-shaped, homogeneous systems 0.46 mm in diameter and 6 mm in length. Each contained 600 µg CGK012 (Figure 4A). In the in vitro release test, 27% of CGK012 was released from PLGA implants for 64 d in 0.9% saline, and approximately 92% was released for 47 d in 2.0% Tween 80 (Figure 4B). The CGK012 implant intraocular profiles are shown in Figure 4C. The Tmax, Cmax, and AUC of CGK012 in the vitreous humor were 28 d, 173 ng/mL, and 8.79 μg∙h/mL, respectively. The CGK012 concentration reached a plateau at 28–42 days and decayed with a half-life of 14 d without systemic exposure.

### 2.5. CGK012 Implants Inhibit Choroidal Neovascularization in a Rabbit Model

**Figure 5:**
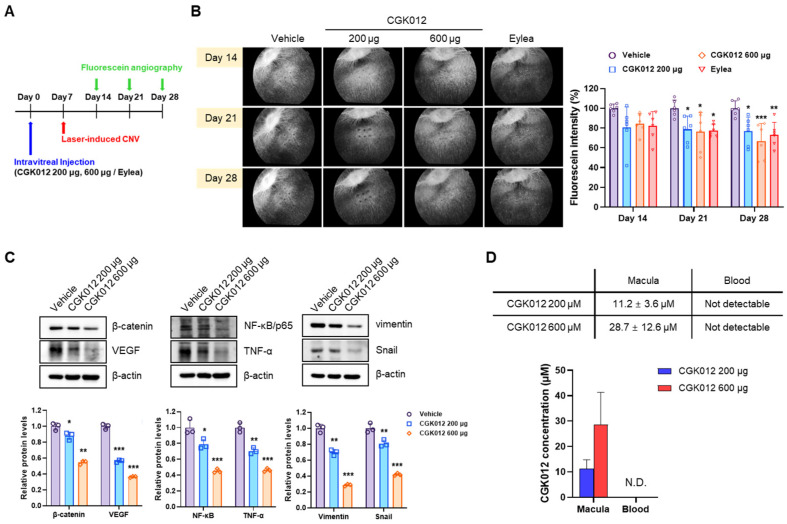
Intravitreal administration of CGK012-loaded PLGA implants inhibits choroidal neovascularization. (**A**) Schematic of the study design. (**B**) Representative images of fluorescein angiography after intravitreal injection of CGK012-loaded PLGA implants into laser-induced CNV chinchilla rabbits. The bar graph represents the intensity of fluorescein. The results represent the mean ± SD (*n* = 6). * *p* < 0.05; ** *p* < 0.01 and *** *p* < 0.001, compared with the vehicle CNV group. (**C**) Whole-cell lysates were prepared from the retina and RPE-choroid of CGK012 treated-CNV rabbits using RIPA buffer. Levels of β-catenin, VEGF, NF-κB/p65, TNF-α, vimentin, or snail were detected using Western Blot. β-actin was used as the loading control. The levels of protein expression were normalized to β-actin. The bar graphs represent the relative intensity of the quantified band, and the results are presented as the mean ± SD of three independent experiments. * *p* < 0.05; ** *p* < 0.01 and *** *p* < 0.001, compared with the vehicle control group. (**D**) Macula and plasma concentrations of CGK012 on Day 28. The results represent the mean ± SD. N.D.: not detected.

Next, we used a laser-induced CNV rabbit model to investigate the CGK012 implants’ in vivo therapeutic effects. When CGK012 implants (200 μg and 600 μg) were administered intravitreally, the area of CNV quantified by fluorescein intensity was notably lower in rabbits treated with CGK012 implants (*n* = 6) compared with controls (*n* = 6) at 14 d (80.68 ± 80.64% and 84.64 ± 9.93%, respectively), 21 d (78.83 ± 13.29% and 76.35 ± 19.41%, respectively), and 28 d (76.86 ± 14.25% and 66.94 ± 17.69%, respectively) (Figure 5A,B). Eylea (Aflibercept), a recombinant decoy VEGF receptor widely used as the standard therapy for nAMD, was employed as a positive control. Markedly, CGK012 implants demonstrated non-inferiority to Eylea for reducing fluorescein intensities at 28 d (66.94 ± 17.69% for CGK012 implants (600 μg) vs. 73.32 ± 12.57% for the Eylea group). We also determined pathogenic factor levels in retina-RPE-choroidal tissue to confirm the inhibitory effect of CGK012 on the Wnt/β-catenin pathway. In agreement with the cell study results, β-catenin, VEGF, NF-κB, and TNF-α levels were decreased in retina-RPE-choroid tissues from rabbits intravitreally injected with CGK012 implants compared with those from control rabbits (Figure 5C). Additionally, Western Blot analysis demonstrated that CGK012 decreased the EMT marker vimentin [16] in laser-induced CNV rabbits (Figure 5C). These results suggest that CGK012 implants suppressed CNV by inhibiting the Wnt/β-catenin pathway. We determined CGK012 concentrations in the macular area and plasma at 28 d after intravitreal administration of CGK012 implants (200 μg and 600 μg). Whereas the plasma concentrations were under the lower limit of quantitation (10 ng/mL), the mean concentrations in the macular area were 11.2 ± 3.6 μM and 28.7 ± 12.6 μM, respectively (Figure 5D), indicating that CGK012 released from PLGA implants was highly permeable to retina/choroid tissue without systemic exposure.

## 3. Discussion

Reports estimate that 14 million patients are blind or nearly blind due to AMD, with nAMD causing approximately 90% of AMD cases with severe vision loss [28]. Since nAMD is a multifaceted disease characterized by multiple pathogenic mechanisms, such as ocular angiogenesis, inflammation, and subretinal fibrosis, therapeutic strategies must be developed to target these different mechanisms. However, until recently, nAMD therapeutics have mainly focused on VEGF and its receptor.

Anti-VEGF therapeutics, such as Lucentis^®^, Avastin^®^, and Eylea^®^, target VEGF-A isoforms and effectively suppress CNV both in animal models and human patients [29]. Additionally, Delivery of protein tyrosine kinase inhibitors (TKIs) via oral or topical ocular routes decreases CNV in animal models by inhibiting the downstream signaling pathways activated by tyrosine kinases of angiogenic receptors [30,31].

The abnormal activation of the Wnt/β-catenin pathway is associated with the progression of AMD. Therefore, inhibiting this pathway may represent a viable strategy for developing new therapeutics. Intravitreal injection of DKK-1 ameliorates retinal inflammation and neovascularization [32]. Additionally, a monoclonal anti-LRP6 antibody (Mab2F1) efficiently reduces vascular leakage and the neovascular area in laser-induced CNV models [15]. Furthermore, nanoparticle delivery of the very-low-density lipoprotein receptor extracellular domain as a Wnt inhibitor via intravitreal injection ameliorates retinal and corneal neovascularization [33]. Although antibody-based therapies are effective, they have limitations, particularly the high costs associated with their manufacturing processes. To address these issues, several studies have investigated small-molecule inhibitors that target the Wnt/β-catenin pathway. Ilimaquinone [34], SERPINA3K [35], curcumin [36], and FH535 [37] have shown the capacity to suppress angiogenesis and fibrotic signaling. However, most findings are derived from in vitro assays or short-term laser-induced CNV models. Furthermore, optimized ocular delivery strategies for long-acting therapies and detailed pharmacokinetic profiles remain insufficient.

In this study, CGK012 suppressed the Wnt/β-catenin pathway, which plays pathogenic roles in nAMD. We demonstrated that in RPE cells and a laser-induced CNV rabbit model, CGK012 lowered VEGF levels and proinflammatory factor expression and blocked the EMT, a key driver of subretinal fibrosis in nAMD. Additionally, CGK012 inhibited capillary tube formation in HUVECs and reduced vascular leakage in the rabbit model for 28 d by attenuating the Wnt/β-catenin pathway.

Anti-VEGF therapeutics are highly effective for nAMD treatment; however, they are limited by their temporary efficacy and requirement for frequent intravitreal injections. Due to these limitations, novel technologies and therapeutics are needed to provide longer-acting nAMD treatment therapies. Biodegradable polymer-based ocular implants represent a feasible new sustained-release drug delivery strategy for nAMD. For example, EYP1901 provides sustained ocular vorolanib release, is a TKI that binds to all VEGFRs, has a biodegradable Durasert E platform, and exhibited a therapeutic duration of six months in human clinical trials [38,39]. Axpaxli, a PEG hydrogel-based intravitreal implant, gradually releases the TKI axitinib over 8–9 months in individuals with nAMD until implant biosorption [40]. In this study, CGK012 was loaded into the biodegradable polymer PLGA, approved by the US Food and Drug Administration in 1997, to prepare intravitreal implants.

The substance is poorly water-soluble, so a surfactant such as Tween 80 is needed to dissolve the compound in physiological solutions. In addition, the implant contains PLGA, the same composition as the dexamethasone intravitreal implant Ozurdex^®^. Therefore, we used the in vitro dissolution protocol following a slight modification [41]. In the in vitro dissolution test, CGK012 dissolved by about 27% over 64 d in normal saline. However, when 2% Tween 80 was added, CGK012 was almost entirely released from the implant in about 50 d, likely because CGK012 solubility in water is very low, so an appropriate surfactant such as Tween 80 is needed to aid dissolution. The equilibrium solubility of CGK012 in normal saline, with and without 2% Tween 80, at 37 °C was 237 and 585 ng/mL, respectively. Tween 80 increases about 2.5-fold in the water solubility of the substance, which is comparable to the result of the in vitro dissolution test.

The Ozurdex^®^ implant is composed of 30% of 50:50 acid-terminated PLGA (Resomer RG 502H), 10% of 50:50 ester-terminated PLGA (Resomer RG 502), and 60% of dexamethasone. The molecular weights of Resomer RG 502 and 502 H are known to be 7000–17,000 g/mol. In the drug development process, one of the significant issues would be the toxicity of vehicles. We chose the exact PLGA composition for the dexamethasone intravitreal implant, Ozurdex^®^, which has been used in the clinic to address the toxicological concerns of the vehicle. A hot-melt extruder prepared the implant, and the implant stick with a diameter of 0.46 mm was cut at every 15 cm. Both edges (1 cm) of each stick were used to check the chemical content of CGK012, and the stick within 5% of the amount of 1 mg was chosen. The final length of the implant was 6 mm.

During the hot melt extrusion, an active substance is mixed with PLGA at about 100 °C. Therefore, a heat-labile substance can be degraded, and a couple of adducts may be generated depending on its chemical structure. Glycolide and/or lactide of the edge of the PLGA can be covalently bonded to the substance. Therefore, lots of attention has to be paid. The melting point of CGK012 is 268 °C, and there is no possible moiety to bind with glycolide and lactide in terms of its chemical structure. LC-MS/MS was used to assess the chemical stability of CGK012 during implant preparation. The chemical content reflects its thermal stability, and no adducts were found.

To ensure the chemical stability of the final product following the electron-beam sterilization, we also measure its content, 0.6 mg per implant. The beam did not affect the substance. The method is frequently used to sterilize final products. It rarely alters the physicochemical properties of an active pharmaceutical ingredient, unlike a sterile method with ethylene oxide.

After administering the implant into the vitreous humor in rabbits, the CGK012 concentration represented a typical pattern of sustained-release preparations; after an initial slow release of about two weeks, the concentration reached a plateau that was maintained for up to 6 weeks and then gradually decreased. The concentration increased again from 70 to 84 d, but the increase was insignificant due to individual variances, and there was a progressive decrease until 140 d. Meanwhile, the cumulative concentrations in the posterior segment of the eye at week 4 after 200 and 600 μg administration to rabbits were 11.2 μM and 28.7 μM, respectively, showing a difference of about three times, proportional to the administered dose. Considering that the ocular implant’s purpose is to treat AMD occurring in the posterior segment of the eye, the continuous release of the active pharmaceutical ingredient in the vitreous humor and dose-dependent distribution to the posterior tissue reduces the administration frequency, thereby increasing patient convenience and drug development potential. Additionally, no systemic exposure was observed after intraocular administration in rabbits. Therefore, promising results can be expected in ruling out toxicological concerns due to systemic exposure, making this implant advantageous for developing eye implants for local application.

In the Wnt/β-catenin pathway, β-catenin protein turnover is predominantly regulated by its N-terminal phosphorylation, which GSK-3β catalyzes in a destruction complex [42]. When GSK-3β activity is inhibited by Wnt3a-CM, CGK012 continues to promote β-catenin phosphorylation at Ser33/Ser37/Thr41 residues and down-regulate intracellular β-catenin levels, suggesting that GSK-3β is not involved in CGK012-mediated β-catenin phosphorylation and degradation. Our previous study has shown that protein kinase Cα (PKCα) phosphorylates β-catenin at Ser33/Ser37/Thr41, leading to degradation of β-catenin [43]. Notably, decursin, a pyranocoumarin from Angelica gigas, and its derivative CGK062 activate PKCα in vitro [43,44,45]. Additionally, CGK062 promotes PKCα-mediated β-catenin phosphorylation and degradation, implying that CGK012 may regulate the Wnt/β-catenin pathway via PKCα activation. Nevertheless, the mechanism underlying CGK012-mediated β-catenin phosphorylation requires additional investigation.

In this study, six eyes were used per group, which may be relatively small. However, results from a small area of the rabbit eye are promising and demonstrate the pharmacokinetics of CGK012 in the vitreous humor, and compare its efficacy with Eylea. Further evaluation of drug release and efficacy across implant formulations is currently underway in a large number of eyes.

## 4. Materials and Methods

### 4.1. Cell Culture, Transfection, Luciferase Assay, and Chemicals

ARPE-19 and L Wnt-3A cells were acquired from the American Type Culture Collection (ATCC). They were cultured in DMEM containing 10% fetal bovine serum (FBS), 120 μg/mL penicillin, and 200 μg/mL streptomycin. Human umbilical vein endothelial cells (HUVECs) were obtained from Cell Engineering for Origin. L Wnt-3A cells secrete biologically active Wnt-3A protein into the medium. To prepare Wnt3a-conditioned medium (Wnt3a-CM), L Wnt-3A cells were incubated in DMEM supplemented with 10% fetal bovine serum (FBS) for 4 days. After this incubation period, the medium was carefully removed and set aside. Fresh medium was then added, and the cells were cultured for an additional 3 days. After this second culture period, the first and second media were combined in a 1:1 ratio and filtered through a 0.22 µm filter. Transfection was conducted using Lipofectamine 2000 (Thermo Fisher, Invitrogen, Waltham, MA, USA) following the manufacturer’s instructions. A luciferase assay was performed using the Dual Luciferase Assay Kit (Promega, Madison, WI, USA) according to the manufacturer’s instructions.

CGK012 was synthesized as previously reported [22] (Figure 1A). In summary, (+)-decursinol (1 equivalents) was added to a solution containing cyclopentyl isocyanate (2.25 Eq), triethylamine (1.8 Eq), and 4-(dimethylamino)pyridine (0.6 Eq) in dry methylene chloride, and the reaction mixture was agitated at 40 °C for 24 h. After evaporating the solvent under reduced pressure, the residue was purified by silica gel column chromatography using a mixture of n-hexane and ethyl acetate in a 2:1 ratio to obtain CGK012[(7S)-(+)-cyclopentyl carbamic acid, 8,8-dimethyl-2-oxo-6,7-dihydro-2H,8H-pyrano [3,2-g]chromen-7-yl-ester] (Figure 1A). The purity of the synthesized CGK012 was determined to be 99.731% using HPLC (Shimadzu Corporation, Kyoto, Japan, detector: 254 nm, column: Shim-pack GIS-ODS (150 × 4.6 mm, 5 μL), column oven: 40 °C, flow rate: 1.0 mL/min, mobile phase: H_2_O: ACN = 55:45). MG132 was obtained from Sigma-Aldrich (St. Louis, MO, USA).

### 4.2. Western Blot

Cytosolic proteins were prepared following the previously described methods [46]. Whole-cell lysates were prepared using RIPA buffer for further analysis. For in vivo studies, rabbits were euthanized 21 days after laser exposure. The eyes of rabbits were immediately isolated, and the neural retina and RPE-choroid segments were separated from the sclera. Tissues were lysed using RIPA buffer, which included a proteinase inhibitor cocktail (Roche, Indianapolis, IN, USA) and phosphatase inhibitor cocktail (Abcam, Cambridge, MA, USA), utilizing a sonicator (Branson Ultrasonics, Danbury, CT, USA). 30 μg of proteins were analyzed by SDS-PAGE and transferred to a nitrocellulose membrane (Bio-Rad, Hercules, CA, USA). The membranes were incubated at room temperature for 1 h in SuperBlock Blocking Buffer (Thermo Scientific, Waltham, MA, USA), followed by overnight incubation at 4 °C with primary antibodies. The primary antibodies used were as follows: anti-β-actin (A1978, 1:2000; Sigma, St. Louis, MO, USA), anti-β-catenin (610154, 1:1000; BD Transduction Laboratories, San Jose, CA, USA), anti-active (non-phospho)-β-catenin (#8814, 1:1000; Cell Signaling Technology, Danvers, MA, USA), anti-E-cadherin (#4065, 1:1000; Cell Signaling Technology), anti-vimentin (sc-7557, 1:1000; Santa Cruz Biotechnology, Dallas, TX, USA) and anti-fibronectin (sc-8422, 1:1000; Santa Cruz Biotechnology). Anti-VEGF (A5708, 1:1000), anti-NF-κB (p65) (A2547, 1:1000), anti-TNF-α (A0277, 1:1000), anti-IL-6 (A0286, 1:1000), anti-IL-1β (A16288, 1:1000), anti-Snail (A5544, 1:1000), α-SMA (A17910, 1:1000), anti-c-myc (A19032, 1:1000), and anti-cyclin D1 (A19038, 1:1000) were purchased from Abclonal (Woburn, MA, USA). Membrane washing was conducted using Tris-buffered saline with Tween 20. The blots were then incubated with anti-mouse immunoglobulin G (IgG)-HRP (sc-516102, 1:1000; Santa Cruz Biotechnology) or anti-rabbit IgG-HRP (sc-2357, 1:1000; Santa Cruz Biotechnology). The bands were detected using the ECL system (sc-2048; Santa Cruz Biotechnology) and analyzed with ImageJ software (NIH, Bethesda, MD, USA).

### 4.3. Scratch Assay

After the ARPE-19 cells were cultured until they formed a confluent monolayer, scratches were created using a pipette tip in the center of the well. After removing cell debris with PBS washing, Wnt3a-CM was added to the cells with CGK012. Images were taken at 48 h and 72 h after scratch induction and examined for the relative cell-free area.

### 4.4. Capillary Tube Formation Assay

HUVEC tube formation was determined in Cultrex reduced growth factor basement membrane extract (BME) (R&D Systems, Minneapolis, CA, USA). Pipette 150 μL of BME into each well of a 48-well plate and incubate at 37 °C for 30 min to allow polymerization to occur. HUVECs were seeded at a density of 3 × 10^4^ cells per well, stimulated with VEGF (10 ng/mL), and treated with CGK012 for 24 h. Tube formation was observed under a light microscope, and the vessel lengths and number of nodes were quantitatively analyzed with ImageJ software.

### 4.5. CGK012-Loaded PLGA Implant Preparation

Two types of PLGA were used for implant preparation: Resomer^®^ RG 502 (Poly(D,L-Lactide-co-Glycolide) lactide to glycolide ratio of 50:50, ester termination) and Resomer^®^ RG 502 H (Poly(D,L-lactide-co-glycolide) acid termination). These two PLGAs, along with CGK012, were mixed in a ratio of 3:1:6 and gently ground in a mortar. Then, 5 g of the mixture was placed into the small-scale twin-screw hot-melt extruder (MIN-CTW, Haake, Berlin, Germany) at 95 °C. Following mixing for 10 min at 50 rpm, the mixture was extruded to form long rods with a diameter of 0.46 mm. The implants were cut to 2 and 6 mm long and inserted into an applicator. After final packing, the preparation was sterilized with electron beam irradiation.

### 4.6. In Vitro Dissolution Test

CGK012 PLGA implants were placed into 50 mL plastic tubes. 50 mL normal saline was added with and without 2% Tween 80. The capped tubes were placed in a shaking water bath at 37 °C, and agitated at a speed of 250 rpm. At predetermined intervals, 45 mL of release medium was withdrawn and replaced with fresh solution. The samples were filtered with a 0.45-μm membrane, and the flow rates were analyzed by high-performance liquid chromatography with tandem mass spectrometry (HPLC-MS/MS) [47].

### 4.7. Animals

All animal care and experimental procedures were performed according to the ARVO statement for the Use of Animals in Ophthalmic and Vision Research. The protocol was approved by the Animal Care and Use Committee of Chung-Ang University (202401030053) and Onheal Co., Ltd. (Onheal Co., Ltd., Incheon, Republic of Korea, 24-HB-0286).

### 4.8. CGK012-Loaded PLGA Implant Administration and Vitreous Sampling

NZW rabbits were given a single vitreous injection (using a special implant syringe with a needle diameter of 1.1 mm) to administer the CGK012 implants. For the positive control group, 2 mg of Eylea (40 mg/mL, 50 μL) was intravitreally injected after induction of choroidal neovascularization by laser. The rabbits were anesthetized, and lidocaine eye drops were given to reduce local pain. The local conjunctiva was cleaned using norfloxacin eye drops and normal saline. The sterilized implants were positioned near the tip of the needle in the syringe for implantation, which then penetrated the sclera approximately 3.5 mm outside the corneoscleral limbus in the superior nasal quadrant of the eye. Next, the needle bar was carefully advanced until the implants were completely delivered into the vitreous cavity. Norfloxacin ophthalmic solutions were used to protect the eyes from infection following the administration of the implant. Vitreous samples (20 μL) were collected from the implanted eye at predetermined intervals after implant injection. The samples were stored at −20 °C until analysis.

### 4.9. Laser-Induced CNV Rabbit Model

The CNV rabbit model, induced by laser photocoagulation, was utilized in studies of exudative AMD [48]. Male chinchilla rabbits (3 months, 2.5 kg) were acquired from Dreambio Co. (Seoul, Republic of Korea) and acclimatized for 1 week. The animals were housed in a room with controlled temperature (23 ± 3 °C), relative humidity (55 ± 15%), illumination intensity (150–300 lux), air ventilation (10–20 times/h), and a 12 h illumination cycle (08:00–20:00). To generate the laser-induced CNV model, the rabbits were anesthetized, and 1% Mydriacyl was applied topically to their right eye to dilate the pupil. Six laser burns were created at about 6 o’clock position, centered on the optic nerve head, with a laser photocoagulator (Elite, Luminis, San Diego, CA, USA) under the conditions of 532 nm, 150 mW, 100 ms.

### 4.10. Fluorescein Angiography and Neovascular Area Quantification

On days 14, 21, and 28 after administering the CGK012-loaded PLGA implant, 1% midriacyl was instilled into the eye of the CNV rabbit model. Subsequently, 1 mL of 2% fluorescein sodium solution was injected into the ear vein, and then fundus fluorescein angiography images were obtained using a fundus camera (TRC-50IX, TOCON, Tokyo, Japan). Fluorescence intensity was analyzed using ImageJ, and the average intensity was calculated.

### 4.11. CGK012 Plasma and Macular Concentration

Blood samples (0.2 mL) were collected weekly from the marginal ear vein of the rabbits for up to 28 d and centrifuged at 12,000× *g* for 5 min. The eyeballs were enucleated under anesthesia right after blood sampling on D 28, and the tissue around the macular area was isolated. The samples were stored at −20 °C until analysis. CGK012 plasma and tissue concentrations CGK012 were determined with HPLC-MS/MS. Briefly, the tissues were homogenized in a threefold excess of PBS (0.1 M, pH 7.4). 90 µL acetonitrile was added to 30 μL plasma and tissue homogenate. The mixtures were stirred vigorously for 10 s and then centrifuged at 12,000× *g* for 5 min. 5 µL supernatant was injected into an HPLC-MS/MS. The HPLC-MS/MS system (API 4000 LC/MS/MS system; AB SCIEX, Framingham, MA, USA) was fitted with an electrospray ionization interface to quantify the compound CGK012. CGK012 was separated with a reversed-phase column (Poroshell EC-C18, 50 × 2.1 mm internal diameter, 2.7 μm particle size; Agilent, Cork, Ireland) at a temperature of 30 °C. The mobile phase consisted of a mixture of distilled water and acetonitrile in a 3:7 (*v*/*v*) ratio with 0.1% formic acid added. This mixture was eluted at a flow rate of 0.2 mL/min using an HP 1260 series pump (Agilent, Wilmington, DE, USA) with a run time of 4 min. The mass transition from precursor to product ions for CGK012 [M + H]+ occurred at *m*/*z* 358.2→229.2. All analytical data were processed using Analyst software (v.1.5.2; Applied Biosystems, Foster City, CA, USA). CGK012 plasma and tissue concentrations CGK012were calculated with calibration graphs prepared from 10 to 1000 ng/mL.

### 4.12. Data and Statistical Analysis

All animals were randomly allocated to either control or treatment groups, each consisting of the same number of animals. The data analysis and quantifications were conducted in a blinded manner to ensure objectivity. The experimental results are presented as mean ± SD. One-way ANOVA was used to compare group differences using Prism 7.04 (GraphPad Software Inc., San Diego, CA, USA), and post hoc analysis was performed using Dunnett’s multiple-comparison test. Statistical significance was monitored at α = 0.05.

## 5. Conclusions

We demonstrated that a PLGA-based intravitreal implant loaded with CGK012, a specific small-molecule inhibitor targeting the Wnt/β-catenin pathway, inhibited CNV development in a laser-induced rabbit model of AMD. CGK012 has a unique mechanism of action by reducing angiogenic/proinflammatory factor expression, suppressing the EMT, and inhibiting capillary tube formation of vascular endothelial cells. Moreover, this implant has the potential to improve convenience, safety, and cost-effectiveness compared to existing anti-VEGF therapies.

## Data Availability

The original contributions presented in the study are included in the article, and further inquiries can be directed to the corresponding authors.
